# Supplementary material for: Prediction of the Sex-Associated Genomic Region in Tunas (Thunnus Fishes)
Source: Int J Genomics. 2021 Dec 14;2021:7226353. doi: 10.1155/2021/7226353 (PMC8693018; doi:10.1155/2021/7226353)
Supplement: Supplementary 1 — Supplementary Table 1: protein-coding genes predicted in scaffold M175. Supplementary Table 2: list of genes used for phylogenetic analysis. [file 7226353.f1.docx]

**Supplementary Table 1. Protein-coding genes predicted in scaffold M175**

| No. | Start | End | Strand | Match in Ensembl | Identity (%) | Description |
| --- | --- | --- | --- | --- | --- | --- |
| g1 | 9390 | 9818 | + | ENSTRUP00000067783 | 41.3 | novel gene |
| g2 | 16389 | 17603 | - | ENSTRUP00000068256 | 63.7 | novel gene |
| g3 | 32614 | 64628 | + | ENSORLP00000007539 | 83.3 | estrogen sulfotransferase (*sult1st6*) |
| g4 | 76852 | 77963 | + | ENSTRUP00000072273 | 69.9 | novel gene |
| g5 | 82490 | 82919 | + | ENSTRUP00000067783 | 37.8 | novel gene |
| g6 | 118954 | 119841 | + | ENSORLP00000040498 | 83.8 | novel gene |
| g7 | 168127 | 168456 | + | ENSORLP00000044417 | 63.3 | novel gene |
| g8 | 176645 | 176974 | + | ENSORLP00000044417 | 63.3 | novel gene |
| g9 | 186243 | 186572 | + | ENSORLP00000044417 | 63.3 | novel gene |
| g10 | 195859 | 196188 | + | ENSORLP00000044417 | 62.4 | novel gene |
| g11 | 200940 | 201269 | + | ENSORLP00000044417 | 63.3 | novel gene |
| g12 | 207325 | 207654 | + | ENSORLP00000044417 | 65.1 | novel gene |

Note: the protein sequence of g2 was matched to ENSTRUP00000068256 (ENSTRUG00000032453, novel gene), with the Pfam domain “PiggyBac transposable element-derived protein,” and g5 was matched to ENSTRUP00000067783 (ENSTRUG00000032600, novel gene) with the Pfam domain “Reverse transcriptase/retrotransposon-derived protein, RNase H-like domain.” Notably, g1 was similar to g5 and matched to ENSTRUP00000067783, suggesting that g2–g4 were sandwiched between duplicated genes (g1 and g5). In addition, g6 was matched to ENSORLP00000040498 (ENSORLG00000025541, novel gene) with the Pfam domain “Transposase, Tc1-like,” and the other genes, g7–g12, were matched to the same protein, ENSORLP00000044417 (ENSORLG00000028531, novel gene) with the Gene3D domain “Immunoglobulin-like fold.”

**Supplementary Table 2. List of genes used for phylogenetic analysis.**

| Species | Gene ID^a^ | Protein ID^a^ | Gene name annotated | Chromosome or scaffold | Location (start..end) | Microsynteny^b^ |
| --- | --- | --- | --- | --- | --- | --- |
| *Acanthochromis polyacanthus* | ENSAPOG00000021511 | ENSAPOP00000033137 | *sult1st6* | MVNR01002684 | 44576..57560 | *pelo - ppp1cab - atp2a1l -* ***sult1st6*** *- mto1* |
| *Amphilophus citrinellus* | ENSACIG00000014524 | ENSACIP00000018748 | *sult1st6* | CCOE01000086 | 2450163..2460056 | *paqr4b - pelo - ppp1cab - atp2a1l -* ***sult1st6*** *- mto1 - adprh* |
| *Amphiprion ocellaris* | ENSAOCG00000023558 | ENSAOCP00000031703 | *sult1st6* | NXFZ01000321 | 131853..142492 | *paqr4b - pelo - ppp1cab - atp2a1l -* ***sult1st6*** *- mto1* |
| *Amphiprion percula* | ENSAPEG00000011124 | ENSAPEP00000015618 | *sult1st6* | chromosome 19 | 27090048..27097145 | *paqr4b - pelo - ppp1cab - atp2a1l -* ***sult1st6*** *- mto1 - adprh* |
| *Anabas testudineus* | ENSATEG00000019443 | ENSATEP00000028139 | *sult1st6* | OOHO01000022 | 8935804..8941421 | *paqr4b - pelo - ppp1cab - atp2a1l -* ***sult1st6*** *- mto1 - adprh* |
| *Astatotilapia calliptera* | ENSACLG00000010044 | ENSACLP00000014735 | *sult1st6* | chromosome 3 | 33234998..33263850 | *paqr4b - pelo - ppp1cab - atp2a1l -* ***sult1st6*** *- mto1 - adprh* |
| *Astyanax mexicanus* | ENSAMXG00000033476 | ENSAMXP00000047131 | *sult1st6* | chromosome 4 | 24751434..24766402 | *paqr4b - pelo - ppp1cab - atp2a1l -* ***ENSAMXG00000038655*** *-* ***sult1st6*** *- mto1 - adprh* |
|  | ENSAMXG00000038655 | ENSAMXP00000048691 |  | chromosome 4 | 24773874..24784608 |  |
| *Astyanax mexicanus* Pachon cavefish | ENSAMXG00005023375 | ENSAMXP00005051894 | *sult1st6* | KB871880 | 377346..390097 | *paqr4b - pelo - ppp1cab - atp2a1l -* ***sult1st6*** *- mto1 - adprh* |
| *Betta splendens* | ENSBSLG00000017417 | ENSBSLP00000035652 | *sult1st6* | chromosome 19 | 12414508..12416490 | *paqr4b - pelo - ppp1cab - atp2a1l -* ***sult1st6*** *- mto1 - adprh* |
| *Clupea harengus* | ENSCHAG00000005386 | ENSCHAP00000009072 | *sult1st6* | chromosome 23 | 21339221..21344295 | *paqr4b - pelo - ppp1cab - atp2a1l -* ***sult1st6*** *- mto1 - adprh* |
| *Cottoperca gobio* | ENSCGOG00000016658 | ENSCGOP00000036117 | *sult1st6* | chromosome 19 | 14497408..14504657 | *paqr4b - pelo - ppp1cab - atp2a1l -* ***sult1st6*** *- mto1 - adprh* |
| *Cynoglossus semilaevis* | ENSCSEG00000008665 | ENSCSEP00000013442 | *sult1st6* | chromosome 8 | 3288519..3302059 | *ppp1cab - pelo - paqr4b - atp2a1l -* ***sult1st6*** *- mto1* |
| *Cyprinodon variegatus* | ENSCVAG00000015871 | ENSCVAP00000028671 | *sult1st6* | KL652975 | 422294..439683 | *paqr4b - pelo - ppp1cab - atp2a1l -* ***sult1st6*** *- mto1 - adprh* |
| *Cyprinus carpio* German mirror | ENSCCRG00010024594 | ENSCCRP00010058073 | *sult1st6* | SAUK01000566 | 584211..588825 |  |
| *Cyprinus carpio* Hebao red | ENSCCRG00020038720 | ENSCCRP00020083996 | *sult1st6* | SAUJ01033560 | 212811..246819 |  |
| *Danio rerio* | ENSDARG00000006811 | ENSDARP00000023637 | *sult1st6* | chromosome 12 | 15197857..15205087 | ***sult1st6*** *- adprh - mto1* |
| *Denticeps clupeoides* | ENSDCDG00000002450 | ENSDCDP00000004929 | *sult1st6* | chromosome 2 | 2949152..2953814 | *pelo - paqr4b - atp2a1l -* ***sult1st6*** *- mto1 - adprh* |
| *Echeneis naucrates* | ENSENLG00000020877 | ENSENLP00000049602 | *sult1st6* | chromosome 19 | 8792821..8796940 | *paqr4b - pelo - ppp1cab - atp2a1l -* ***sult1st6*** *- mto1 - adprh* |
| *Electrophorus electricus* | ENSEEEG00000022646 | ENSEEEP00000048166 |  | RBHW02000133 | 290539..295671 | *paqr4b - pelo - ppp1cab - atp2a1l -* ***ENSEEEG00000022646*** *-* ***sult1st6*** *- mto1 - adprh* |
|  | ENSEEEG00000022677 | ENSEEEP00000048175 | *sult1st6* | RBHW02000133 | 302430..308876 |  |
| *Esox lucius* | ENSELUG00000023121 | ENSELUP00000041980 | *sult1st6* | LG05 | 32291062..32316287 | *paqr4b - pelo - ppp1cab - atp2a1l -* ***ENSELUG00000023158*** *-* ***sult1st6*** *- mto1 - adprh* |
|  | ENSELUG00000023158 | ENSELUP00000024398 |  | LG05 | 33298188..33336211 |  |
| *Fundulus heteroclitus* | ENSFHEG00000009934 | ENSFHEP00000026853 | *sult1st6* | KN805689 | 39665..70498 | *paqr4b - pelo - ppp1cab - atp2a1l -* ***sult1st6*** *- mto1* |
| *Gambusia affinis* | ENSGAFG00000019360 | ENSGAFP00000028649 | *sult1st6* | NHOQ01002480 | 608781..616381 | *paqr4b - pelo - ppp1cab - atp2a1l -* ***sult1st6*** *- mto1 - adprh* |
| *Gasterosteus aculeatus* | ENSGACG00000007411 | ENSGACP00000009844 | *sult1st6* | groupV | 9510733..9515238 | *paqr4b - pelo - ppp1cab - atp2a1l -* ***sult1st6*** *- mto1 - adprh* |
| *Gouania willdenowi* | ENSGWIG00000024469 | ENSGWIP00000050304 | *sult1st6* | chromosome 19 | 190193..201093 | *paqr4b - pelo - ppp1cab - atp2a1l -* ***sult1st6*** |
| *Haplochromis burtoni* | ENSHBUG00000005742 | ENSHBUP00000004463 | *sult1st6* | JH425862 | 364970..376082 | *paqr4b - pelo - ppp1cab - atp2a1l -* ***sult1st6*** |
| *Hippocampus comes* | ENSHCOG00000017445 | ENSHCOP00000026410 |  | KV879664 | 359333..362372 | *paqr4b - pelo - ppp1cab - atp2a1l -* ***sult1st6*** *-* ***ENSHCOG00000017236*** *-* ***ENSHCOG00000017445*** *- mto1 - adprh* |
|  | ENSHCOG00000017236 | ENSHCOP00000026337 |  | KV879664 | 362963..368150 |  |
|  | ENSHCOG00000017153 | ENSHCOP00000013738 | *sult1st6* | KV879664 | 366333..373628 |  |
| *Hucho hucho* | ENSHHUG00000021597 | ENSHHUP00000034261 | *sult1st6* | QNTS01000080 | 830931..869034 | *pelo -* ***sult1st6*** *- mto1* |
| *Ictalurus punctatus* | ENSIPUG00000020293 | ENSIPUP00000030029 |  | chromosome 13 | 25897128..25907337 | *paqr4b - pelo - ppp1cab - atp2a1l -* ***sult1c1*** *- mto1- adprh -* ***ENSIPUG00000020306*** *-* ***ENSIPUG00000020293*** |
|  | ENSIPUG00000020306 | ENSIPUP00000030052 |  | chromosome 13 | 25908803..25923694 |  |
|  | ENSIPUG00000020450 | ENSIPUP00000030270 | *Sult1c1* | chromosome 13 | 26002733..26010057 |  |
| *Kryptolebias marmoratus* | ENSKMAG00000021410 | ENSKMAP00000028880 | *sult1st6* | LWHD01000005 | 8791060..8800264 | *paqr4b - pelo - ppp1cab - atp2a1l -* ***sult1st6*** *- mto1 - adprh* |
| *Labrus bergylta* | ENSLBEG00000022660 | ENSLBEP00000029954 | *sult1st6* | FKLU01000006 | 450363..460517 | *paqr4b - pelo - ppp1cab - atp2a1l -* ***sult1st6*** *- mto1 - adprh* |
| *Larimichthys crocea* | ENSLCRG00005006343 | ENSLCRP00005014839 | *sult1st6* | XVI | 7953919..7959498 | *paqr4b - pelo - ppp1cab - atp2a1l -* ***sult1st6*** *- mto1 - adprh* |
| *Lates calcarifer* | ENSLCAG00010008143 | ENSLCAP00010017168 | *sult1st6* | CYIF01000522 | 147925..153169 | *paqr4b - pelo - ppp1cab - atp2a1l -* ***sult1st6*** *- mto1 - adprh* |
| *Mastacembelus armatus* | ENSMAMG00000013107 | ENSMAMP00000019480 | *sult1st6* | OOHQ01000019 | 10836988..10845975 | *paqr4b - pelo - ppp1cab - atp2a1l -* ***sult1st6*** *- mto1 - adprh* |
| *Maylandia zebra* | ENSMZEG00005013915 | ENSMZEP00005018595 | *sult1st6* | LG8 | 10704507..10713143 | *paqr4b - pelo - ppp1cab -* ***ENSMZEG00005014201*** *-* ***ENSMZEG00005014130*** *- atp2a1l -* ***sult1st6*** *- mto1 - adprh* |
|  | ENSMZEG00005014130 | ENSMZEP00005018899 |  | LG8 | 10724653..10734055 |  |
|  | ENSMZEG00005014201 | ENSMZEP00005018962 |  | LG8 | 10743124..10762994 |  |
| *Mola mola* | ENSMMOG00000001367 | ENSMMOP00000001653 | *sult1st6* | KV751339 | 5419160..5424872 | *paqr4b - pelo - ppp1cab - atp2a1l -* ***sult1st6*** *- mto1* |
| *Monopterus albus* | ENSMALG00000008317 | ENSMALP00000011703 | *sult1st6* | KV884985 | 237259..255497 | *paqr4b - pelo - ppp1cab - atp2a1l -* ***sult1st6*** *- mto1 - adprh* |
| *Myripristis murdjan* | ENSMMDG00005003142 | ENSMMDP00005005636 | *sult1st6* | chromosome 19 | 10319554..10325912 | *paqr4b - pelo - ppp1cab - atp2a1l -* ***sult1st6*** *- mto1 - adprh* |
| *Neogobius melanostomus* | ENSNMLG00000022670 | ENSNMLP00000036563 | *sult1st6* | VHKM01000605 | 944167..949851 | *paqr4b - pelo - ppp1cab - atp2a1l -* ***sult1st6*** *- adprh* |
| *Neolamprologus brichardi* | ENSNBRG00000011976 | ENSNBRP00000015442 | *sult1st6* | JH422419 | 326786..338419 | *paqr4b - pelo - ppp1cab - atp2a1l -* ***sult1st6*** *- adprh* |
| *Oreochromis aureus* | ENSOABG00000004542 | ENSOABP00000008345 | *sult1st6* | VASH01008598 | 534735..545517 | *paqr4b - pelo - ppp1cab - atp2a1l -* ***sult1st6*** *- mto1 - adprh* |
| *Oreochromis niloticus* | ENSONIG00000011490 | ENSONIP00000014462 | *sult1st6* | LG8 | 16447459..16458774 | *paqr4b - pelo - ppp1cab - atp2a1l -* ***sult1st6*** *- mto1 - adprh* |
| *Oryzias latipes* | ENSORLG00000005995 | ENSORLP00000007539 | *sult1st6* | chromosome 19 | 7537015..7546975 | *paqr4b - pelo - ppp1cab - atp2a1l -* ***sult1st6*** *- mto1 - adprh* |
| *Oryzias latipes* HNI | ENSORLG00020016251 | ENSORLP00020015238 | *sult1st6* | chromosome 19 | 6495878..6506331 | *paqr4b - pelo - ppp1cab - atp2a1l -* ***sult1st6*** *- mto1 - adprh* |
| *Oryzias latipes* HSOK | ENSORLG00015014237 | ENSORLP00015013339 | *sult1st6* | chromosome 19 | 7549755..7556865 | *paqr4b - pelo - ppp1cab - atp2a1l -* ***sult1st6*** *- mto1 - adprh* |
| *Oryzias melastigma* | ENSOMEG00000021293 | ENSOMEP00000019467 | *sult1st6* | NVQA01000018 | 6924507..6936317 | *paqr4b - pelo - ppp1cab - atp2a1l -* ***sult1st6*** *- mto1 - adprh* |
| *Parambassis ranga* | ENSPRNG00000010056 | ENSPRNP00000019269 | *sult1st6* | chromosome 19 | 11920057..11923483 | *paqr4b - pelo - ppp1cab - atp2a1l -* ***sult1st6*** *- adprh* |
| *Paramormyrops kingsleyae* | ENSPKIG00000008115 | ENSPKIP00000025134 |  | PGUA01000716 | 77339..85158 | ***sult1st6*** *-* ***ENSPKIG00000008115*** *- mto1* |
|  | ENSPKIG00000008157 | ENSPKIP00000025227 | *sult1st6* | PGUA01000716 | 89460..92302 |  |
| *Periophthalmus magnuspinnatus* | ENSPMGG00000014573 | ENSPMGP00000018010 | *sult1st6* | KN465639 | 159687..165096 | *paqr4b - pelo - ppp1cab -* ***sult1st6*** *- mto1 - adprh* |
| *Poecilia formosa* | ENSPFOG00000006629 | ENSPFOP00000006618 | *sult1st6* | KI519918 | 462869..470226 | *paqr4b - pelo - ppp1cab - atp2a1l -* ***sult1st6*** *- mto1 - adprh* |
| *Poecilia mexicana* | ENSPMEG00000017160 | ENSPMEP00000014751 | *sult1st6* | KQ551698 | 216029..226910 | *paqr4b - pelo - ppp1cab - atp2a1l -* ***sult1st6*** *- mto1 - adprh* |
| *Poecilia reticulata* | ENSPREG00000023085 | ENSPREP00000034085 | *sult1st6* | LG19 | 8606073..8612537 | *paqr4b - pelo - ppp1cab - atp2a1l -* ***sult1st6*** *- adprh* |
| *Pundamilia nyererei* | ENSPNYG00000017166 | ENSPNYP00000022724 | *sult1st6* | JH419445 | 551287..561679 | *paqr4b - pelo - ppp1cab - atp2a1l -* ***sult1st6*** *- mto1 - adprh* |
| *Pygocentrus nattereri* | ENSPNAG00000023594 | ENSPNAP00000017338 |  | KV576018 | 205036..217798 | *atp2a1l -* ***sult1st6*** *-* ***ENSPNAG00000023594*** *- mto1 - adprh* |
|  | ENSPNAG00000023544 | ENSPNAP00000034252 | *sult1st6* | KV576018 | 221917..230084 |  |
| *Salarias fasciatus* | ENSSFAG00005021044 | ENSSFAP00005042392 | *sult1st6* | chromosome 8 | 14815378..14819495 | *paqr4b - pelo - ppp1cab - atp2a1l -* ***sult1st6*** *- mto1 - adprh* |
| *Salmo salar* | ENSSSAG00000006601 | ENSSSAP00000013233 | *sult1st6* | ssa19 | 60560309..60629821 | *pelo -* ***sult1st6*** *- atp2a1l - mto1 - adprh* |
| *Salmo trutta* | ENSSTUG00000044402 | ENSSTUP00000099012 | *sult1st6* | chromosome 2 | 70656921..70706772 | *pelo -* ***sult1st6*** *- atp2a1l - mto1 - adprh* |
| *Scleropages formosus* | ENSSFOG00015013340 | ENSSFOP00015051479 | *sult1st6* | chromosome 20 | 1696811..1711132 | *pelo -* ***sult1st6*** *- mto1* |
| *Scophthalmus maximus* | ENSSMAG00000017115 | ENSSMAP00000027971 | *sult1st6* | chromosome 18 | 12047529..12053057 | *paqr4b - pelo - ppp1cab -* ***sult1st6*** *- atp2a1l - mto1 - adprh* |
| *Seriola dumerili* | ENSSDUG00000022981 | ENSSDUP00000031995 | *sult1st6* | BDQW01000068 | 11264858..11272211 | *paqr4b - pelo - ppp1cab - atp2a1l -* ***sult1st6*** *- mto1 - adprh* |
| *Seriola lalandi dorsalis* | ENSSLDG00000017784 | ENSSLDP00000022796 | *sult1st6* | PEQF01098412 | 74658..80011 | *paqr4b - pelo - ppp1cab - atp2a1l -* ***sult1st6*** *- mto1 - adprh* |
| *Sinocyclocheilus anshuiensis* | ENSSANG00000040067 | ENSSANP00000080667 | *sult1st6* | LAVE01S001531 | 871785..912247 |  |
| *Sinocyclocheilus grahami* | ENSSGRG00000019146 | ENSSGRP00000034664 | *sult1st6* | LCYQ01S002242 | 32340..69243 |  |
| *Sinocyclocheilus rhinocerous* | ENSSRHG00000001387 | ENSSRHP00000001967 | *sult1st6* | LAVF01S023966 | 141242..191906 |  |
| *Sparus aurata* | ENSSAUG00010025659 | ENSSAUP00010063865 | *sult1st6* | chromosome 20 | 14038665..14044473 | *paqr4b - pelo - ppp1cab - atp2a1l -* ***sult1st6*** *- mto1 - adprh* |
| *Sphaeramia orbicularis* | ENSSORG00005024862 | ENSSORP00005055782 | *sult1st6* | chromosome 19 | 32726559..32733416 | *paqr4b - pelo - ppp1cab - atp2a1l -* ***sult1st6*** *- mto1 - adprh* |
| *Stegastes partitus* | ENSSPAG00000008291 | ENSSPAP00000010893 | *sult1st6* | KK581727 | 122862..134768 | *paqr4b - pelo - ppp1cab - atp2a1l -* ***sult1st6*** *- mto1 - adprh* |
| *Takifugu rubripes* | ENSTRUG00000023795 | ENSTRUP00000052023 | *sult1st6* | chromosome 1 | 12680685..12683854 | *paqr4b - pelo - ppp1cab - atp2a1l -* ***sult1st6*** *- mto1 - adprh* |
| *Tetraodon nigroviridis* | ENSTNIG00000018940 | ENSTNIP00000022128 | *sult1st6* | chromosome 2 | 7791682..7794346 | *paqr4b - pelo - ppp1cab - atp2a1l -* ***sult1st6*** *- mto1 - adprh* |
| *Xiphophorus maculatus* | ENSXMAG00000018534 | ENSXMAP00000023350 | *sult1st6* | chromosome 10 | 4086676..4099611 | *paqr4b - pelo - ppp1cab - atp2a1l -* ***sult1st6*** *- mto1 - adprh* |
| ^a^IDs in the Ensembl release 99. |  |  |  |  |  |  |
| ^b^*sult1st6* are shown in bold. Unannotated intervening genes are ignored. | |  |  |  |  |  |
