# Supplementary material for: Prediction of the Sex-Associated Genomic Region in Tunas (Thunnus Fishes)
Source: Int J Genomics. 2021 Dec 14;2021:7226353. doi: 10.1155/2021/7226353 (PMC8693018; doi:10.1155/2021/7226353)

**List of supplementary figures**

**Supplementary Figure 1.** Comparison between PBT (*T. orientalis*) male scaffolds and SBT (*T. maccoyii*) male chromosomal regions. The upper panel indicates the alignment between PBT scaffold M44 and a part of SBT chromosome 4 (1–6,000,000 bp) (accession no. NC_056536). The lower panel indicates the alignment between PBT scaffold M175 and a part of SBT chromosome 13 (2,500,000–2,800,000 bp) (accession no. NC_056545). In M175, PBT male’s haploid region (18,001–78,750 bp) detected by CNV analysis is highlighted in gray.

**Supplementary Figure 2.** Comparison between the pseudo-haploid scaffolds obtained from Supernova2 and scaffolds M44/M175. The upper panel indicates the alignment between the Supernova2 scaffolds (scaffold IDs: 91818, 90643, 93129, and 275) and M44. The lower panel indicates the alignment between the Supernova2 scaffolds (scaffold IDs: 52814, 81188, 87065, 86196, 90712, 81419, 81140, 88535, 91688, and 82315) and M175. In the panels, SLR_M44_ and SLR_M175_ are highlighted in gray.

**Supplementary Figure 3.** Mapped read depths around SLRs in 31 resequenced PBT samples. For each of the samples, SLR is highlighted in gray.

**Supplementary Figure 4.** Genome-wide association with the sex of PBT using male masked reference sequences. Manhattan plot (left) and QQ plot (right) are shown. In the Manhattan plots, genome-wide significance cutoffs based on Bonferroni correction are indicate by a red line.

**Supplementary Figure 5.** Comparison between male and female scaffolds encoding *sult1st6a*. The position of *sult1st6a* in scaffold M30 is indicated by an arrow.

**Supplementary Fig. 1**


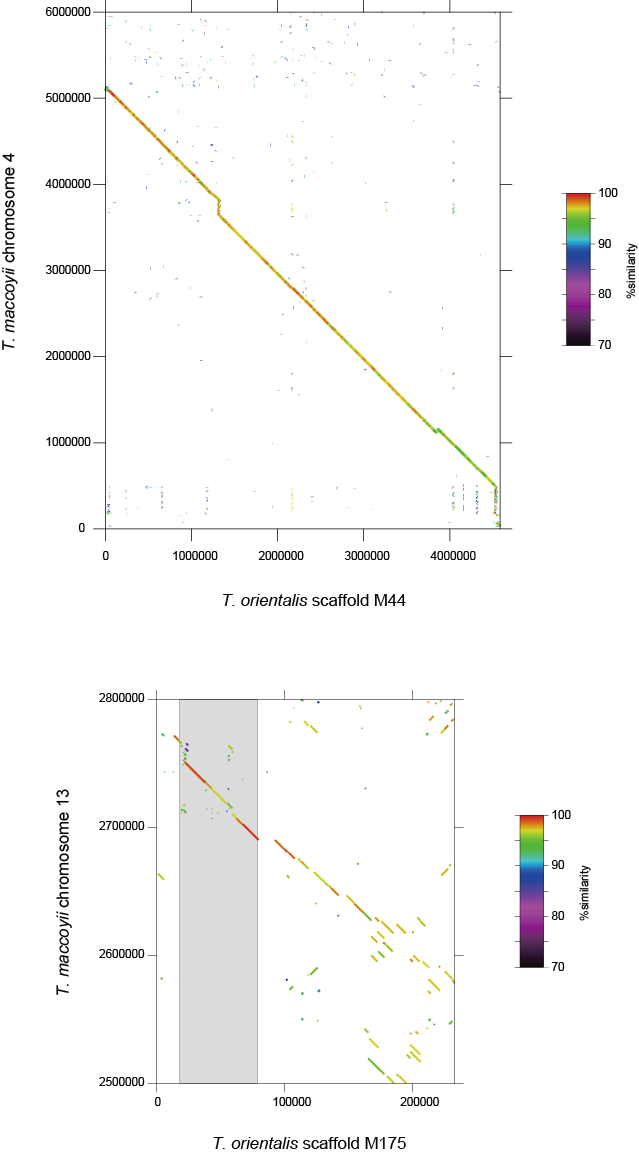


**Supplementary Fig. 2**


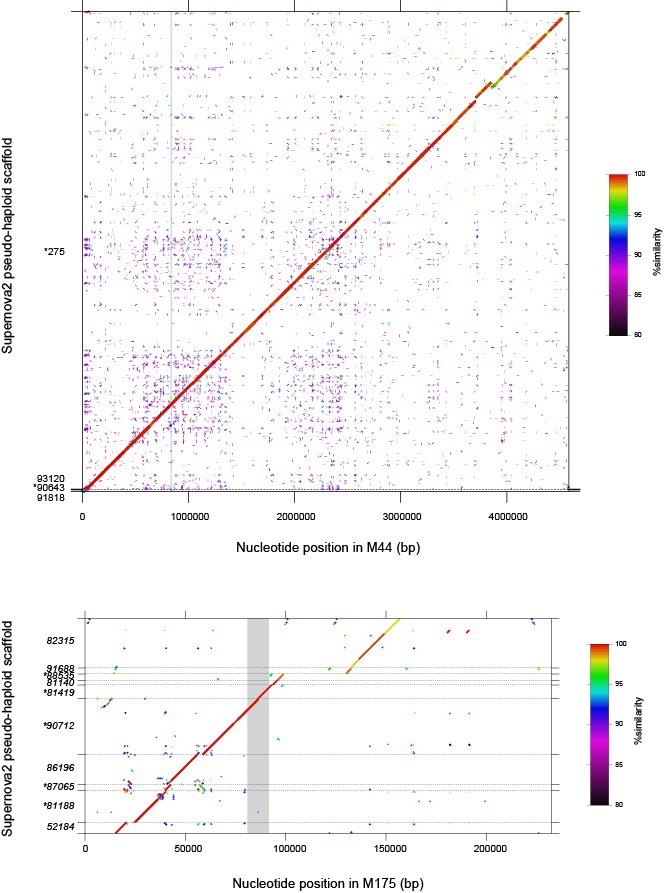


**Supplementary Fig. 3**

**Supplementary Fig. 4**

**Supplementary Fig. 5**


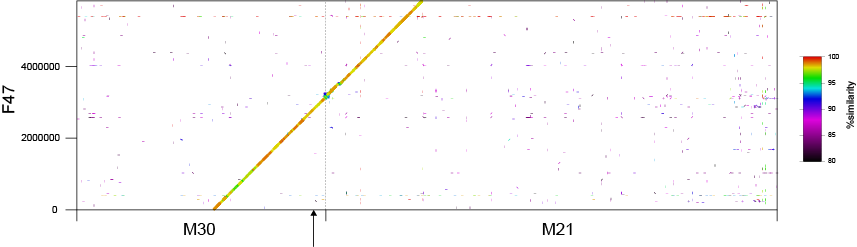

Supplement: Supplementary 2 — Supplementary Figure 1: comparison between PBT male scaffolds and SBT male chromosomal regions. Supplementary Figure 2: comparison between the pseudo-haploid scaffolds obtained from Supernova2 and scaffolds M44/M175. Supplementary Figure 3: mapped read depths around SLRs in 31 resequenced PBT samples. Supplementary Figure 4: genome-wide association with the sex of PBT using male masked reference sequences. Supplementary Figure 5: comparison between male and female scaffolds encoding sult1st6a. [file 7226353.f2.docx]
